# Diagnosis, Phenotype, and Molecular Genetics of Congenital Analbuminemia

**DOI:** 10.3389/fgene.2019.00336

**Published:** 2019-04-17

**Authors:** Lorenzo Minchiotti, Gianluca Caridi, Monica Campagnoli, Francesca Lugani, Monica Galliano, Ulrich Kragh-Hansen

**Affiliations:** ^1^Department of Molecular Medicine, University of Pavia, Pavia, Italy; ^2^Laboratory of Molecular Nephrology, Istituto Giannina Gaslini (IRCCS), Genoa, Italy; ^3^Department of Biomedicine, Aarhus University, Aarhus, Denmark

**Keywords:** analbuminemia, autosomal recessive, frequency, preterm birth, hyperlipidemia, compensatory mechanisms, DNA-sequencing, pathogenic variations

## Abstract

Congenital analbuminemia (CAA) is an inherited, autosomal recessive disorder with an incidence of 1:1,000,000 live birth. Affected individuals have a strongly decreased concentration, or complete absence, of serum albumin. The trait is usually detected by serum protein electrophoresis and immunochemistry techniques. However, due to the existence of other conditions in which the albumin concentrations are very low or null, analysis of the albumin (*ALB*) gene is necessary for the molecular diagnosis. CAA can lead to serious consequences in the prenatal period, because it can cause miscarriages and preterm birth, which often is due to oligohydramnios and placental abnormalities. Neonatally and in early childhood the trait is a risk factor that can lead to death, mainly from fluid retention and infections in the lower respiratory tract. By contrast, CAA is better tolerated in adulthood. Clinically, in addition to the low level of albumin, the patients almost always have hyperlipidemia, but they usually also have mild oedema, reduced blood pressure and fatigue. The fairly mild symptoms in adulthood are due to compensatory increment of other plasma proteins. The condition is rare; clinically, only about 90 cases have been detected worldwide. Among these, 53 have been studied by sequence analysis of the *ALB* gene, allowing the identification of 27 different loss of function (LoF) pathogenic variants. These include a variant in the start codon, frame-shift/insertions, frame-shift/deletions, nonsense variants, and variants affecting splicing. Most are unique, peculiar for each affected family, but one, a frame-shift deletion called Kayseri, has been found to cause about one third of the known cases allowing to presume a founder effect. This review provides an overview of the literature about CAA, about supportive and additional physiological and pharmacological information obtained from albumin-deficient mouse and rat models and a complete and up-to-date dataset of the pathogenic variants identified in the *ALB* gene.

## Introduction

Congenital analbuminemia (CAA; OMIM # 616000) is an autosomal recessive disorder. In homozygous or compound heterozygous persons the trait leads to the complete absence or to strongly decreased concentrations of serum albumin. CAA often results in increased morbidity and mortality during pregnancy and early childhood. However, if affected persons are able to compensate to a sufficient degree the many properties and functions of albumin, they can survive without having the protein and live fairly normal lives (Minchiotti et al., 2013; The Albumin website, 2018).

Human serum albumin (ALB; OMIM # 103600) is synthesized by liver hepatocytes and continuously secreted into the blood. The rate of synthesis and secretion in a healthy, adult person is ca. 14 g per day, and the half-life of ALB is ca. 19 days (Peters, 1996). The total amount in such a person is ca. 360 g, of which about two-thirds are outside the bloodstream and about one-third is in the bloodstream. However, the concentration of ALB is higher in the blood (35–45 g/L), it accounts for 60–65% of total protein in plasma and contributes with ca. 80% of the oncotic pressure of plasma (ca. 15 mm of Hg) (Peters, 1996). This pressure helps keeping the blood within the circulation. The presence of many acidic and basic amino acid residues implies that the protein has an important buffering capacity. ALB has more acidic (98 Glu + Asp) than basic (83 Lys + Arg) residues resulting in a net charge of ca. -15 at physiological pH, a fact that renders the protein important for the Donnan effect in the capillaries. Finally, if necessary, ALB can serve as a source of amino acids or energy (Kragh-Hansen, 2016).

The ability of ALB to bind ligands, and thereby to function as an important depot and transport protein for numerous endogenous and exogenous compounds, is well-known (Kragh-Hansen, 2016). However, ALB also seems to be the quantitatively most important circulating antioxidant, and it has significant and useful enzymatic properties (Kragh-Hansen, 2013). Finally, the protein has anti-inflammatory effects and contributes positively to endothelial stabilization and to the maintenance of the normal capillary permeability (Spinella et al., 2016).

ALB is synthesized as a single polypeptide chain without prosthetic groups and covalently bound lipid or carbohydrate (Peters, 1996). The globular protein consists of 585 amino acids, and it has a molecular mass of 66.5 kDa. The three-dimensional structure of ALB, without or with bound ligand, has been determined in several laboratories, and now the structure is known to a resolution of 2.3 Å (Kragh-Hansen, 2016). It has been found to be a predominantly α-helical heart-shaped molecule, which consists of three homologous domains, each of which is composed of two subdomains that possess common structural motifs.

The ALB is a member of a group of binding proteins called the albumin superfamily (Kragh-Hansen et al., 2013). ALB is the quantitatively most important component of this group, because its plasma concentration is ca. 600 μM, whereas that of the vitamin D-binding protein (Gc-globulin) and afamin (α-albumin) is only ca. 5 and 0.8 μM, respectively. A fourth member of the superfamily, α-fetoprotein, is practically speaking absent in healthy, adult persons, but it is an important plasma protein in the fetal state. Genetic variants of all four proteins have been found, and vitamin D-binding protein is the most polymorphic (Kragh-Hansen et al., 2013). The fifth gene of this superfamily, named the α-fetoprotein related gene, has been found in primates, but in human have several pathogenic variants lead to an inactive pseudogene (Naidu et al., 2010). Finally, recent phylogenetic and structural analyses have revealed that extracellular matrix protein 1 (ECM1) also belongs to this superfamily (Li et al., 2017).

**FIGURE 1:**
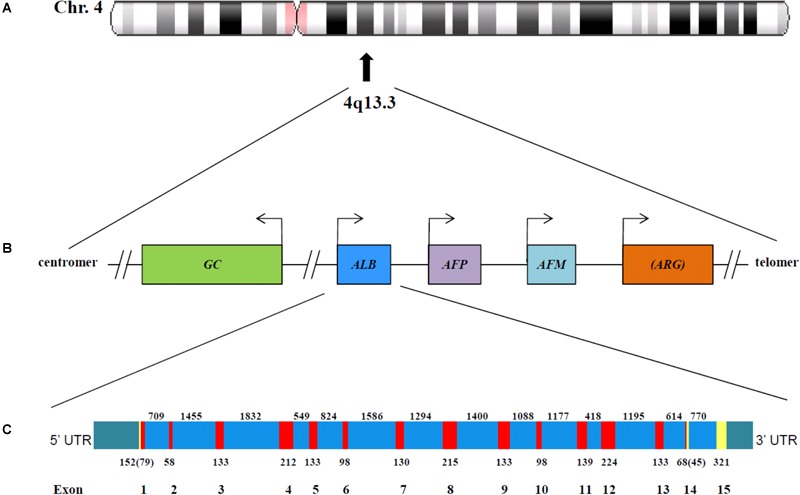
The chromosomal localization **(A)** and genomic organization **(B)** of five of the six albumin superfamily genes. The gene for ECM1 is located at 1q21.3. The arrows represent the start of transcription. The figure is constructed using information published by Speeckaert et al. (2006) and Naidu et al. (2010) and found in the GenBank (http://www.ncbi.nlm.nih.gov/pubmed). *GC*, vitamin D-binding protein (GenBank Gene ID: 2638); *ALB*, albumin (GenBank Gene ID: 213); *AFP*, α-fetoprotein (GenBank Gene ID: 174); *AFM*, afamin (GenBank Gene ID: 1731); *ARG*, α-fetoprotein related gene, which is inactive in primates. As shown, the genes for *ALB, AFP*, and *AFM* are tandemly arranged in the same transcriptional orientation; *ARG* is also oriented in the same way but it is in parenthesis, because in humans it is an inactive pseudogene (Naidu et al., 2010). Whether the reverted orientation of *DBP* has a regulatory or other function has not yet been clarified. **(C)** Genomic structure of the human albumin gene. The linear map of the gene indicating the location of exons (red) and introns (blue) is constructed on the basis of information found in GenBank. The partially untranslated regions of exons 1 and 14, and the completely untranslated exon 15, are in yellow, and the 5′ and 3′ untranslated region UTR are in green. The numbers below the exons indicate the exon size (in bp); numbers in parentheses for exons 1 and 14 represent the number of coding nucleotides. The number between exons represent intron length (in bp).

The gene for ALB (*ALB*; NCBI Genomic Sequence: NC_00004.12) is a single autosomal gene, which is situated at position 4q13.3 near the centromere of chromosome 4 (Minghetti et al., 1986) (Figure 1A). The single-copy genes of vitamin D-binding protein, afamin, α-fetoprotein and the α-fetoprotein related gene, are also placed in this part of the chromosome (Figure 1B). The five genes have arisen from a common ancestor through a series of duplication events and are tightly linked in humans and in all other species studied. The relation between these genes and the one for ECM1 has not yet been clarified, but the latter is located on a completely different genomic context, namely at position 1q21.3 (GenBank^1^). The four, first-mentioned, genes are expressed in a co-dominant manner.

The *ALB* gene is 16,961 nucleotides long from the putative “cap” site to the first poly(A) addition site (Minghetti et al., 1986). As seen from Figure 1C, the gene is divided by 14 intervening introns into 15 exons, the last of which is untranslated. The exons are symmetrically placed in such a way that they correspond to the three domains of the protein. It has been proposed that the domains have arisen by triplication of a single primordial domain (Minghetti et al., 1986).

The messenger ribonucleic acid (mRNA) for ALB (NCBI Reference Sequence NM_000477.6) encodes a precursor protein called preproalbumin which consists of 609 amino acids (NCBI Reference Sequence: NP_000468.1). Hydrolysis from the N-terminal end of the signal peptide of 18 amino acids and of the propeptide of six amino acids results in the mature protein of 585 amino acids (Peters, 1996).

The *ALB* gene shows a significant degree of DNA polymorphism. To date, 73 nucleotide substitutions (mainly missense) have been reported to cause a circulating variant of ALB or of its proprotein (The Albumin Website, 2018). In its heterozygous form, this condition is known as alloalbuminemia or bisalbuminemia (OMIM # 103600). The genetic variants do not seem to be associated with disease, neither in the heterozygous nor in the homozygous form. Only the variants resulting in familial dysalbuminemic hyperthyroxinemia and hypertriiodothyroninemia are of clinical relevance, because affected individuals are at risk of inappropriate treatment or may have adverse drug effects (Kragh-Hansen et al., 2017). In 27 other examples, the pathogenic variants (mainly affecting splicing, nonsense, and deletions) cause a premature stop in the ALB synthesis and lead to the condition known as CAA (The Albumin Website, 2018; Caridi et al., 2019). In this review, we discuss the diagnosis, differential diagnoses, frequency and clinical impact of CAA. Information obtained by albumin-deficient rats and mice will be mentioned. Finally, the 27 currently known variants of *ALB* will be tabulated and shortly described in molecular terms.

## Diagnosis of and Differential Diagnosis to CAA

**FIGURE 2:**
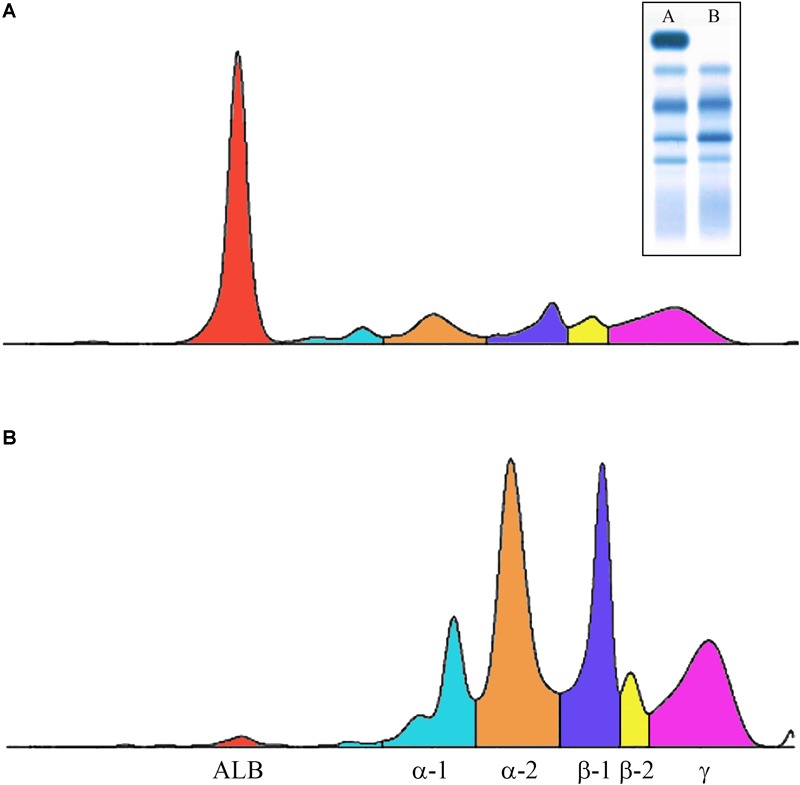
Capillary electrophoresis of serum proteins. The profiles were obtained via the fully automated Helena V8 Capillary Electrophoresis System. **(A)** Normal; **(B)** patient having the Erzurum trait (Caridi et al., 2016a). Inset: conventional serum protein electrophoresis (A, normal; B, analbuminemic subject). Both types of electrophoresis allow to detect that in the patient ALB is near absent, whereas all the globulin fractions are increased.

Congenital analbuminemia is an inherited condition characterized by the absence, or by an abnormally low level, of ALB in the blood serum (Figure 2) (Minchiotti et al., 2013). Many methods are commonly used in clinical chemistry laboratories to establish the ALB concentration: conventional or capillary serum protein electrophoresis, standard clinical chemistry systems using photometric dye-binding methods, and immunochemistry techniques (Caridi et al., 2018a). Of these, dye-binding assays overestimate ALB levels at low concentrations (Lyon et al., 1998), to the point where concentrations around or greater than 15 g/L have been recently reported in analbuminaemic subjects (Caridi et al., 2018a,b, 2019). This, together with the absence of unambiguous clinical and biochemical signs in the analbuminaemic individuals (see below), may represent difficulties in the early clinical diagnosis of CAA. In contrast, immunonephelometric techniques in association with serum protein electrophoresis (Figure 2), represent probably the most accurate method to test a well-founded suspicion of CAA on biochemical bases, giving ALB levels < 1 g/L in analbuminaemic persons (Caridi et al., 2018a).

In addition to CAA, several other inherited syndromes result in low ALB concentrations. Congenital nephrotic syndrome is such a genetic disorder, and several genes have been implicated in its etiology (Bérody et al., 2019). The estimated cumulative incidence in France is 0.5 per 100,000 live births, but the incidence of the syndrome has been reported to be as high as 1 per 8,000 live births in Finland (Bérody et al., 2019). Congenital protein losing enteropathy results in a low ALB level due to protein-losing enteropathy and severe diarrhea (Stephen et al., 2016). The enteropathy is the result of an inborn error of lipid metabolism caused by a variant in the *DGAT1* gene. The prevalence of the disease is estimated to ca. 1 per 300,000, but the condition is most probably underestimated (Stephen et al., 2016). Normally, the intracellular neonatal Fc receptor, FcRn, which resides in the endosomes, binds and thereby prevents ALB from degradation in the lysosomes. Instead the protein is exocytosed from the cells, a process which provides a long half-life of the protein. However, in familial hypercatabolic hypoproteinemia FcRn is deficient. This situation results in an increased catabolism of ALB and in a marked reduction of the protein serum levels (Wani et al., 2006). Finally, early onset ataxia with ocular motor apraxia also shows hypoalbuminemia (Yokoseki et al., 2011). The causative variants for this disorder are found in the aprataxin gene.

Decreased ALB levels can also be caused by diseases, which are not inherited (Minchiotti et al., 2013). Thus, low ALB concentrations can be caused by many, more common disorders such as renal or intestinal loss (glomerular nephritis, nephrotic syndrome, and protein losing gastroenteropathy), redistribution into extravascular compartments (septicaemia and other inflammatory states) and insufficient production rate (severe hepatic cirrhosis).

In conclusion, although protein electrophoresis of serum samples (Figure 2) and immunochemistry techniques are well-suited screening methods for the potential detection of CAA, the wide range of the measured ALB levels, the absence of clear clinical and biochemical evidence, and the fact that hypoalbuminemia can be caused by many, often more common, disorders represent major pitfalls in the early clinical diagnosis of CAA. Therefore, genetic examination of the *ALB* is mandatory to establish the diagnosis of CAA.

## Frequency of CAA

Congenital analbuminemia is a very rare condition, since its prevalence has been estimated to less than 1:1,000,000 live births, apparently without gender or ethnicity predilection (Peters, 1996). After the first case of a 31-years old German woman reported in 1954 (Bennhold et al., 1954), only about 90 cases are so far described worldwide with 27 different variants (Table 1), most of which are recorded in the Register of Analbuminemia Cases (The Albumin website, 2018).

**Table 1:** Table of analbuminemia causing variants.

#^a^	Variant name^b^	Intron/exon	dbSNP (#rs)	Nucleotide change^c^	Protein change^d^	Reference (the first report describing the variant)	Number of cases
1	Afula	E-1		c.1A>C	Undefined defect	Caridi et al., 2013	2 (closely related) families
2	Baghdad	I-1	rs77408163	c.79+1G>A	Undefined splicing defect	Campagnoli et al., 2002	1
3	Madeira	I-2		c.138-2A>G	Undefined splicing defect	Caridi et al., 2018a	1
4	Codogno	E-3	rs75470261	c.166C>T	p.Gln56Ter	Watkins et al., 1994b	1
5	Kayseri	E-3	rs75152012	c.228_229del	p.Val78Cysfs^∗^2	Galliano et al., 2002	14
6	Amasya	E-3		c.229_230del	p.Val78Cysfs^∗^2	Caridi et al., 2012a	1
7	Treves	I-3		c.270+1G>T	Undefined splicing defect	Caridi et al., 2016b	1
8	Bethesda	E-4	rs77238412	c.412C>T	p.Arg138Ter	Watkins et al., 1994b	2
9	Erzurum	E-5		c.527del	p.Pro176Argfs^∗^65	Caridi et al., 2016a	2 (same family)
10	Zonguldak	E-5	rs281860282	c.597T>A	Undefined splicing defect?	Caridi et al., 2008	1
11	Nijmegen-2	E-5	rs773532821	c.615G>A	Undefined splicing defect	Unpublished	1
12	Vancouver	I-6	rs77335374	c.714-2A>G	Undefined splicing defect	Ruffner and Dugaiczyk, 1988	1
13	Seattle	E-7	rs76454301	c.714G>A	p.Trp238Ter	Watkins et al., 1994b	1
14	El Jadida	E-7	rs78340021	c.802G>T	p.Glu268Ter	Campagnoli et al., 2005b	2
15	Roma	E-8	rs77449454	c.872dup	p.Asn291Lysfs^∗^8	Watkins et al., 1994a	2 (same family)
16	Bologna	E-8		c.920del	p.Leu307Argfs^∗^23	Dagnino et al., 2010b	1
17	Ghazaouet	E-9		c.1098dup	p.Val367fs^∗^12	Caridi et al., 2019	2 (same family)
18	Roma-2	E-10		c.1225C>T	p.Gln409Ter	Campagna et al., 2005	1
19	Monastir	E-10	rs281860283	c.1275C>A	p.Tyr425Ter	Caridi et al., 2009	1
20	Guimarães	I-10	rs779988470	c.1289+1G>A	p.Phe398Alafs^∗^33	Caridi et al., 2012b	2 + 2 (same family)
21	Fondi	E-11	rs398089012	c.1427A>G	p.Tyr476Serfs^∗^13	Campagna et al., 2005	1
22	Tripoli	I-11		c.1428+1G>T	Undefined splicing defect	Bibi et al., 2012	2 (same family)
23	Bartin	I-11	rs78784172	c.1428+2T>C	p.Leu431Tyrfs^∗^5	Dolcini et al., 2007	1
24	Tubingen	E-12	rs78696173	c.1525C>T	p.Arg509Ter	Ruhoff et al., 2010	2 (same family)
25	Locust Valley	E-12	rs77081291	c.1610del	p.Ile537Asnfs^∗^21	Davis et al., 2008	1
26	Safranbolu	E-12		c.1614_1615del	p.Leu540Phefs^∗^2	Dagnino et al., 2010a	2
27	Ankara	I-12		c.1652+1G>A	p.Leu477Cysfs^∗^4	Caridi et al., 2014	1

*^*a*^The variants are ordered on the basis of their positions in the albumin gene. ^*b*^The variants have been named after the place from where the first detected carrier originates. ^*c*^bp and codon numbering is according to HGVS rules and is based on the cDNA sequence NM_000477.6. ^*d*^The protein changes in analbuminemia are deduced from mRNA (Guimarães, Fondi, Bartin, and Ankara) or DNA (all the others) sequence and were not established at the protein level. Subtract 24 from amino acid numbers to convert to starting at the Asp1 of mature albumin.*

The frequency of the trait can be much higher in restricted and minimally admixed population groups than in the average population. The most significant example are two First Nations communities of Cree descent living in the North-western central plains regions in Saskatchewan (Canada) (Meyer, 1984). Anthropological studies of this region report that between 1870 and 1960 the population was primarily sustained by within group marriages, suggesting that the relatively elevated incidence of CAA in this population group is due to a founder effect (Meyer, 1984). Similarly, historical marriage patterns by the residents of a Slovak gypsy settlement have likely increased the frequency of homozygous analbuminemic subjects and of heterozygous carriers (Rosipal, personal communication).

During recent years the new technologies, Next Generation Sequencing (NGS), in particular exome sequencing (WES), and genome sequencing (WGS), have transformed and exponentially improved the possibility to identify rare genetic variants in different populations. The NGS has made possible the development of large population-based sequencing databases that are an essential tool to allow the molecular diagnosis of rare diseases. In this review, we obtained data about the *ALB* gene from two large public sequence databases that were collected to catalog the spectrum of genetic variants across the general population: The Genome Aggregation Database (2018) (*n* = 15,496 WGS and *n* = 123,136 WES) and Bravo Database (2018) (by TOPMed Freeze5; *n* = 62,784 WGS). Participants were ascertained from a wide array of family based and case-controlled cohorts and from randomized, controlled trial populations. Both dataset exclude related individuals and cases with pediatric-onset disease present in the index case or in a first-degree family member, but do include participants with adult-onset disease, including diabetes, cardiovascular diseases, and psychiatric disorders. Both databases include participants from multiple ethnicities including European, Finnish, African, South Asian, and Latino.

We selected all the *ALB* variants present in gnomAD and BRAVO; then we annotated them based on a reference sequence (NM_000477.6) and filtered by the web interface only the loss of function (LoF) variants that are usually associated with the CAA phenotype. We identified a total of 19 different *ALB* variants (Tables 2, 3), 5 of them already described (Table 3) and 14 new (Table 2). None of the latter were present in homozygous state, this fact and the low frequency of the heterozygous confirm that the CAA is a rare disorder.

**Table 2:** Table of LoF variants present in public database but not yet identified as cause of CAA.

#^a^	Intron/exon	dbSNP (#rs)	Nucleotide change^b^	Protein change^c^	Allele count/ allele number
1	E-1	rs1213633671	c.8G>A	p.Trp3Ter	1/125568^e^
2	E-1	rs867930468	c.70C>T	p.Arg24Ter	1/246242^d^
3	E-4	rs1250260879	c.282delT	p.Phe94Leufs^∗^47	1/125568^e^
4	E-4	rs1368509385	c.352C>T	p.Gln118Ter	1/30958^d^; 1/125568^e^
5	E-5	rs768570250	c.656_659del	p.Lys219Argfs^∗^21	1/246042^d^
6	E-7	rs1179582806	c.861T>A	p.Tyr287Ter	1/246052^d^
7	E-7	rs762322697	c.901_908del	p.Glu301Ter	1/246146^d^
8	E-7	rs766066361	c.1010del	p.Lys337Argfs^∗^35	1/246132^d^
9	E-8	rs764429360	c.1120del	p.Ala374Profs^∗^8	1/246050^d^
10	E-8	rs958244328	c.1179C>A	p.Cys393Ter	2/276956^d^; 1/125568^e^
11	I-10	rs779988470	c.1289+1G>C	Undefined splicing defect	1/244656^d^
12	I-12	rs1274226966	c.1485_1486insA	p.Arg496Lysfs^∗^24	1/125568^d^
13	I-12	rs77635837	c.1652+2T>A	Undefined splicing defect	1/30964^d^
14	I-12	rs77635837	c.1652+2T>C	Undefined splicing defect	1/244334^d^

*^*a*^Variants are ordered on the basis of their position in the albumin gene. ^*b*^bp and codon numbering are according to HGVS rules and are based on the cDNA sequence NM_000477.6. ^*c*^For the protein changes subtract 24 from amino acid numbers to convert to starting at the Asp1 of mature albumin. ^*d*^Public Dataset gnomAD (version 2.0.2.). ^*e*^Public Dataset Bravo.*

**Table 3:** Table of LoF variants present in public database identified as cause of CAA.

#^a^	Variant name^b^	Intron/exon	dbSNP (#rs)	Nucleotide change^c^	Protein change^d^	Allele count/allele number
1	Baghdad	I-1	rs77408163	c.79+1G>A	Undefined splicing defect	2/246244^e^
2	Kayseri	E-3	rs75152012	c.228_229del	p.Val78Cysfs^∗^2	14/276934^e^; 8/125568^f^
3	Bethesda	E-4	rs77238412	c.412C>T	p.Arg138Ter	2/277026^e^
4	Guimarães	I-10	rs779988470	c.1289+1G>A	p.Phe398Alafs^∗^33	3/275346^e^; 2/125568^f^
5	Fondi	E-11	rs398089012	c.1427A>G	p.Tyr476Serfs^∗^13	1/229286^e^

*^*a*^Variants are ordered on the basis of their position in the albumin gene. ^*b*^Variants have been named after the place, from where the first detected carrier originates. ^*c*^bp and codon numbering are according to HGVS rules and are based on the cDNA sequence NM_000477.6. ^*d*^The protein changes in analbuminemia are deduced from mRNA (Guimarães and Fondi) or DNA (all the others) sequence and were not established at the protein level. Subtract 24 from amino acid numbers to convert to starting at the Asp1 of mature albumin. ^*e*^Public Dataset gnomAD (version 2.0.2). ^*f*^Public Dataset Bravo.*

The cumulative frequency of the five CAA-causing variants reported in the normal population (dataset gnomAD and BRAVO) is 1/6,295 (159 in 1,000,000 individuals), with the Kayseri deletion having the most frequent allele, 1/9,155 (109 in 1,000,000 individuals) (Table 3). The databases also list other 14 molecular defects, which have not yet been identified among the so far known cases of the condition (Table 2), but which might be associated to the disorder, when the number of patients with CAA molecular diagnosis will increase.

A few other deficiencies and challenges in estimating the frequency of CAA should be mentioned. For example, one does not know, how many of the miscarriages are caused by CAA (see section “Consequences of CAA”). In addition, the frequency of CAA in the adult population is uncertain, because establishing the diagnosis of CAA for certain calls for a fairly advanced clinical-biochemical laboratory, which does not always exist in smaller hospitals. Finally, some cases could be unnoticed due to the mild symptoms.

## Consequences of CAA

Congenital analbuminemia can have serious consequences in the pre- and peri-natal period. Thus, CAA in the fetus is an important cause of preterm birth, which is the world’s leading cause of neonatal death (Staps et al., 2018). It has been proposed that the preterm births are caused by oligohydramnions and placental abnormalities (Staps et al., 2018). However, CAA can also in a direct way be a risk factor for the fetus itself during pregnancy and later in childhood. This hypothesis is in accordance with reports describing the death of several individuals in the fetal state or in early infancy (Minchiotti et al., 2013). Actually, CAA has been reported to be the second largest direct cause of child deaths in children younger than 5 years (Staps et al., 2018).

The above-mentioned effects of CAA are based on accidental findings of the trait, but they can be supplemented by a comprehensive and detailed study of Toye et al. (2012) of two First Nations communities living in Saskatchewan, Canada. By searching hospital health records between 2001 and 2009 the authors identified 12 cases of CAA in children of between 1 day and 15 months of age. Two-thirds of the cases were born preterm, two-thirds were of low birth weight, and a quarter were small for their gestational age. Most of the children had oedema or signs of fluid retention as well as lower respiratory tract infections. There were three mortalities in the case series caused by overwhelming infections. The seven mothers have had a total of 40 pregnancies and at least seven miscarriages. Findings like preterm birth, low birth weight, small size, presence of miscarriages in the family history, respiratory distress with frequent hospital admissions and mild developmental delay were also found in more recent studies of single families living in Turkey (Caridi et al., 2014, 2016a) or Germany (Caridi et al., 2016b). In conclusion, all these data suggest increased morbidity and mortality during pregnancy and early infancy in patients with CAA.

In contrast to early in life, if the patient reach adulthood CAA is a relatively benign condition due to compensatory measures. Follow-up information about individuals with CAA is sketchy and not much is known about, for example, their life span. The Albumin website reports that one female died at an age of 32, whereas six other persons lived until they were 55–75 years old. The website also mentions three examples of parenting of children. In this connection, it is relevant to note that Hu et al. (2017) have followed a 24-year-old pregnant woman with CAA of the Kayseri defect, see below. The female has had one previous miscarriage, she was overweight with oedema and lipodystrophy but was otherwise in good health. During the pregnancy she received albumin infusions, deep vein thrombosis prophylaxis and several medications. The treatment ensured that she had an uncomplicated delivery and gave birth to a healthy (heterozygous) live male infant. However, pregnancies are not always successful. Thus, a 37-year-old analbuminemic woman was reported to have serious problems during her first pregnancy (Caridi et al., 2019). She had a pronounced weight gain during the first 2 months of pregnancy (from 96 to 132 kg), and clinical examination showed the presence of multiple oedemas and lipodystrophy. The pregnancy had to be stopped by an emergency caesarean section because of uterine bleeding and hemorrhagic shock (Caridi et al., 2019).

Although CAA in adulthood is a relatively benign syndrome, it leads to several biochemical and health changes. The most obvious one is on the concentration of plasma proteins, because ALB is very low or completely absent. However, the compensatory increase in other plasma proteins, which partly can take-over the lacking ALB functions, reduce the potential severity of the symptoms. The quantitative most important increase is seen for the globulins, but the concentration is also increased in the case of other non-albumin proteins such as lipoproteins, transferrin, α_1_-antitrypsin, thyroxin binding globulin and coagulation factors. As a result, the total serum protein level is only slightly decreased in most analbuminemic individuals. Systemic and pronounced oedema is not seen, because oedema-preventive mechanisms are operative such as mitigation of the effect on the oncotic gradient and lowering of the hydrostatic blood pressure gradient from the capillaries to the interstitial fluids as well as a decrease in the capillary permeability of proteins (Koot et al., 2004). Despite the compensatory measures, CAA is usually associated with mild oedema, reduced blood pressure and fatigue.

Congenital analbuminemia has a great impact on the lipid metabolism resulting in gross hyperlipidemia with severe hypercholesterolemia and elevated LDL-cholesterol levels. In more detail, increased esterified cholesterol, free fatty acids and apolipoprotein B concentrations may occur as well as increased high-density lipoprotein-3 and apolipoprotein A-I and A-II levels (Crook, 2016). By contrast, the levels of HDL-cholesterol and triglycerides are usually normal. The severe hypercholesterolemia may be the result of a compensatory mechanism for the lack of ALB and provides evidence for the possible role of ALB in controlling lipoprotein metabolism (Crook, 2016). Some adult patients, especially females, develop a peculiar lower body lipodystrophy, often with abnormal body habitus (Peters, 1996; Minchiotti et al., 2013). In extreme cases the lipodystrophy requires leg liposuction and controlled compression therapy (Kandamany and Munnoch, 2014). Somewhat surprisingly, the risk of osteoporosis seems to be increased. It is not known, whether the hyperlipidemia leads to premature atherosclerosis or to the onset of still other diseases (Caridi et al., 2018a). However, the severe hypercholesterolemia observed in most analbuminaemic individuals may have been responsible for some cases of premature coronary heart disease (Demirsoy et al., 2011).

The low concentrations, or even total absence, of ALB compromises its ability to function as a transport and depot protein for a range of endogenous ligands. The important transport of free fatty acids is partly taken over by an increased concentration of apolipoprotein B-100. The bilirubin binding capacity in serum has been reported to be only 25% of normal capacity. However, perinatal jaundice in newborns has not been described (Koot et al., 2004). Also the binding capacity for inorganic ions can be affected. This results in, for example, hypocalcemia, because the protein-bound pool of the metal ion is decreased. However, often the concentration of unbound, active calcium ions is normal (Koot et al., 2004).

A challenge in the CAA research is the problem of follow-up studies of patients. Such studies would be very valuable for seeing the long-term effects of the metabolic changes associated with the trait.

Clinical trials on the efficacy of ALB infusions in patients with CAA have demonstrated a transitory decrease in cholesterol and a variety of plasma proteins. However, no long-term benefit of the therapy has been found. Furthermore, it is not practical to infuse CAA patients with ALB at regular intervals over their lifetime, because of the cost of intravenous ALB and its limited potential to affect outcome (Newstead et al., 2004). In addition, ALB preparations for clinical use are still isolated from human blood and therefore could contain infectious agents. However, albumin therapy for a shorter period can be relevant, for example during pregnancy (Hu et al., 2017) or in connection with surgery (Demirsoy et al., 2011).

Patients with CAA are often treated with drugs, which are directed against sequela of their condition or against other types of health problems. For example, in many cases the hypercholesterolemia has been treated with cholesterol-lowering drugs such as simvastatin or atorvastatin. However, other medications can also be relevant, and many of the commonly used drugs are highly albumin-bound. The unbound fraction of this type of drugs is usually higher than expected; for example, 10-fold increases in the free fractions of warfarin and diazepam have been reported (Hu et al., 2017). Therefore, albumin-binding drugs should be used with caution and monitored carefully, mainly because of earlier onset of side-effects and toxicity.

## Albumin-Deficient Animal Models

For obtaining useful physiological and pharmacological information of relevance for analbuminemic persons, investigations have been performed with albumin-deficient mouse and rat models. For example, Roopenian et al. (2015) have created knock-out mouse models with disruption of the *Alb* mouse gene; one of the models had also the mouse *FcgRn* gene replaced by the human version. The animals were analbuminemic but healthy, and they bred similarly to their parental strains. Administration of human ALB to the mice possessing the human FcRn gene had a serum half-life of ca. 24 days, whereas the half-life was only 2.6–5.8 days in mice without the human version of the FcRn gene. These findings stress species differences in FcRn and the importance of the receptor in the recycling, and thereby the half-life, of ALB. Roopenian et al. (2015) also found that the analbuminemic mice with mouse *FcgRn* had small but significant reductions in total serum protein as compared to mice having both the *Alb* and the *FcgRn* gene (from 5.8/5.6 to 3.7/3.8 g/dL). These results show that even though the synthesis of non-albumin serum proteins were increased, they could not fully compensate for the lacking Alb. Interestingly, the mouse models with disruption of the *Alb* gene showed no tendency to oedema formation. Total triglycerides, cholesterol, LDH and lipase activity were increased, whereas the concentration of non-esterified fatty acids were decreased. Finally, total bilirubin and iron were decreased, but calcium, alanine aminotransferase and aspartate aminotransferase were elevated.

Some of the above findings were also made in Nagase analbuminemic rats (Lee et al., 2013). Nagase analbuminemic rats are natural mutant Sprague-Dawley rats, which do not express Alb due to a single splice variant in the albumin gene. In an attempt to compensate for the lacking Alb, the concentrations of especially β- and γ-globulins are increased in the Nagase rats. The accompanying hyperlipidemia is due to increased cholesterol and triglyceride concentrations. Interestingly, the plasma volume was larger in the Nagase rats than in control rats. In addition, Mani et al. (2012) found that Nagase rats have bradycardia compared with wild type rats due to increased production of nitric oxide. Apparently, it is not known whether a similar phenomenon exists in humans. For additional effects of the analbuminemic condition in Nagase rats, see the monograph of Peters (1996).

In contrast to the above-mentioned mouse models, Nagase rats have been used to study the pharmacokinetics of drugs normally bound to Alb and other plasma proteins. For example, Lee et al. (2013) report that a large number of drugs (e.g., furosemide, azosemide, bumetanide, torasemide, omeprazole, gliclazide, and warfarin) are weakly bound to plasma proteins in Nagase rats, and that their total body clearance are increased by 170% to more than 1,000%. Toyama et al. (2014) found that intravenous administration of sunitinib also had increased systemic clearance in analbuminemic rats. This observation was partly due to decreased plasma protein binding and increased volume of distribution at steady state. Finally, according to Yoshimura et al. (2015) the total amount of mycophenolic acid in plasma after intravenous injection was much lower in Nagase rats than in controls. However, the free fraction was increased. The Nagase rats showed a higher total body clearance than the controls did. In this example the volume of distribution of the drug was smaller than in the wild type animal. In contrast, inactivation of the drug by glucuronidation were not significantly different between the Nagase rats and controls.

Thus, the information obtained with animal models largely confirms and supplements the findings in analbuminemic humans. An advantage of using the analbuminemic rodents is that it is easier to examine the pharmacokinetics and pharmacodynamics of plasma protein-binding drugs and albumin-based therapeutics in detail.

## Molecular Genetics

### Types of CAA-Causing Variants

**FIGURE 3:**
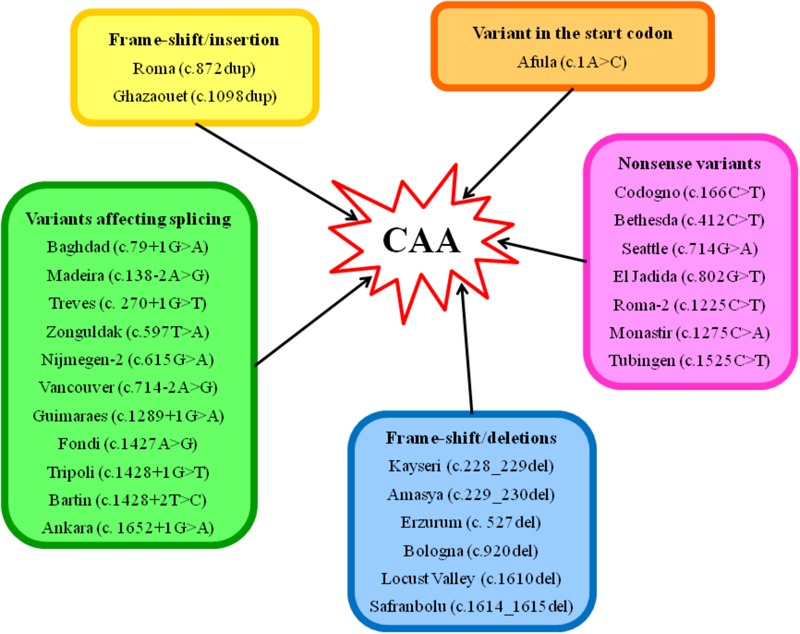
Scheme of the type of variants, which are known to cause CAA in humans. The molecular defects are named after the place from where the first detected carrier originates. Codon numbering is according to HGVS rules and based on the cDNA sequence NM_000477.6.

To date, 70 cases are listed in the Analbuminemia Register (The Albumin Website, 2018). To these must be added about twenty other cases, which are not yet included in the Register due to the lack of adequate records. In particular, 8 additional cases were reported among the children born between 1996 and 2009 in two remote First Nations communities living in the North-western central plains region in Saskatchewan, Canada (Toye et al., 2012). The presence of some older cases in this population group may have been missed (Toye et al., 2012). In addition, a few examples were found among the members of a Slovak gypsy settlement (Rosipal, personal communication). Of the about 90 cases, 53 have been studied at the molecular level. The studies confirmed that CAA is an autosomal recessive disorder, and allowed the identification of 27 different causative defects placed in either the 14 exons of the *ALB* or in intron/exon junctions (Table 1). Only for case #32 of the Register, the cause of CAA could not be found within the *ALB* (unpublished result). Consanguinity was shown to be a factor in most cases in which it was possible to reconstruct the genealogical tree of the affected family. Figure 3 gives a survey of the 27 known defects. Twenty five of the 27 different variants identified in analbuminemic subjects cause CAA at the homozygous state. As seen in Figure 3 and detailed below, they include a variant in the start codon, frame-shift/insertion, frame-shift/deletions, nonsense variants, and variants affecting splicing. Of these possibilities, variants affecting splicing (11 cases), nonsense variants (7 cases), and frame-shift/deletions (6 cases) seem to be the most common causes (Table 1). Compound heterozygosity for two molecular defects, namely a nonsense variant (Roma-2) and a splice site variant with subsequent reading frame-shift (Fondi), caused CAA in an Italian man; case #23 of the Register (Campagna et al., 2005).

### Short Presentation of the Individual Types of Variant

#### Variant in the Start Codon

##### Analbuminemia Afula

A homozygous variant c.1A>C in the start codon was found to cause CAA in two members of two closely related families of the Druze population living in a Galilean village in Northern Israel. This variation causes the loss of the primary start codon ATG for Met1, which is replaced by an untranslated triplet CTG for Leu (p.Met1Leu). The use of an alternative downstream ATG codon would probably give rise to a completely aberrant polypeptide chain. The absence of the pre- and pro-peptide most likely prevents handling of the translation product by the normal secretion pathway, leading to a misrouted intracellular transport and a premature degradation (Caridi et al., 2013).

#### Frame-Shift/Insertions

##### Analbuminemia Roma

A single A insertion in exon 8 in a string of four A residues, c.872dup, was found to cause CAA in an Italian family. The mother was homozygous for the trait, whereas the daughter was heterozygous. This additional base leads to a frame-shift causing a premature stop codon 7 amino acids downstream, giving rise to a putative translation product, p.Asn291Lysfs^∗^8, consisting of 273 residues (Watkins et al., 1994a).

##### Analbuminemia Ghazaouet

Two analbuminemic individuals of a consanguineous family were found to be homozygous for a thymine insertion in exon 9 (c.1098dup). The subsequent frame-shift causes a premature stop codon, giving rise to an aberrant truncated putative protein product, p.Val367fs^∗^12, consisting of 353 amino acids. The same variant is present in heterozygous condition in several other members of the family (Caridi et al., 2019).

#### Frame-Shift/Deletions

##### Analbuminemia Kayseri

The Kayseri variant (c.228_229del), an AT deletion at positions c.228–229 which are the 91st and 92nd bases of exon 3, produces a frame-shift leading to a premature stop, two codons downstream. The predicted translation product (p.Val78Cysfs^∗^2) would consist of only 54 amino acids (Galliano et al., 2002). The Kayseri defect is the most common cause of CAA. So far, it has been found in 14 analbuminemic individuals: two of Amerindian and two of Turkish origin, three Slovak Romany children, members of the same family, a Swedish male, three members of a family of Arab ethnicity living in Israel, a young girl of Punjabi origin living in United Kingdom (Minchiotti et al., 2013), a young Australian woman of Lebanese origin (Berglund et al., 2015) and in an analbuminemic subject of Turkish origin (LM et al., unpublished result). In addition, it is very likely the cause of CAA in at least 10 other members of the First Nation band of Cree origin in the East Central Canadian Province of Saskatchewan (Lyon et al., 1998; Newstead et al., 2004; Toye et al., 2012). Finally, the trait most likely exists in members of a Slovak gypsy settlement (Rosipal, personal communication). However, unfortunately, it was not possible to study these cases at the molecular level.

##### Analbuminemia Amasya

An overlapping TG deletion (c.229_230del) was identified in a young Turkish man, born from a first cousin marriage. This deletion leads to a frameshift that introduces a premature stop codon two amino acids downstream, thus producing a predicted translation product (p.Val78Cysfs^∗^2) of only 54 amino acids (Caridi et al., 2012a). This short polypeptide chain is identical to that predicted as a consequence of the Kayseri defect.

##### Analbuminemia Erzurum

Congenital analbuminemia in two Turkish infant siblings was shown to be caused by a homozygous deletion of a C in exon 5, in a stretch of four C starting from the nucleotide in position 524 and ending at position 527 (c.527del). The sequence of four C in which the deleted base is located includes the last two nucleotides in the codon for Ala175 and the first two bases in the codon for Pro176. The subsequent frame-shift changes the codon for Pro176 to Arg, leading to a premature stop codon TGA at position 240, 65 amino acid residues downstream, near the 5′ end of exon 7 (p.Pro176Argfs^∗^65). Therefore, the predicted translation product from the Erzurum allele would be 215 amino acids long. The consanguineous (first-degree cousins) parents are both heterozygous for the same variation (Caridi et al., 2016a).

##### Analbuminemia Bologna

A single thymine deletion in exon 8 at position c.920 (c.920del) was found to cause CAA in an Italian pediatric patient. The deleted thymine represents the second base in the codon for p.Leu307, and the variation causes a frame-shift, leading to a premature stop codon 22 amino acids downstream (p.Leu307Argfs^∗^23). The predicted translation product from the Bologna allele would therefore consist of 304 residues (Dagnino et al., 2010b). The apparently non-consanguineous parents are both heterozygous for the same variation.

##### Analbuminemia Locust Valley

A single thymine deletion in exon 12 was found to cause analbuminemia Locust Valley (c.1610del) in a paraplegic man of Italian descent. The deletion of thymine from codon p.537 causes a frame-shift with premature termination after the translation of 20 new amino acids. Thus, the predicted truncated variant protein would be 532 amino acids in length, the longest of any ALB molecule associated with CAA. The novel C-terminal sequence, however, contains a proalbumin-like sequence that has the potential to be post-translationally shortened (Davis et al., 2008).

##### Analbuminemia Safranbolu

A CA deletion near the 3′ end of exon 12, at nucleotide positions c.1614–1615 in the codons for p.Cys538 and p.Thr539 (c.1614_1615del) was identified as the cause of CAA in a young Turkish woman of consanguineous parents. The subsequent frame-shift changes the codon for p.Leu540 to Phe and introduces a stop codon TGA at position p.541. The predicted translation product (p.Leu540Phefs^∗^2) would consist of 516 amino acids (Dagnino et al., 2010a). Very recently, we have detected the same deletion in an Italian analbuminemic woman (Suppressa et al., 2019).

#### Nonsense Variants

##### Analbuminemia Codogno

A single-base substitution, C>T at position c.166 in exon 3 (c.166C>T), resulting in the change of the codon CAG for p.Gln56 to stop codon TAG (Watkins et al., 1994b) was found to cause analbuminemia in an Italian male. This extremely premature termination codon would encode an ALB fragment of only 31 amino acids (p.Gln56Ter), the shortest putative product of any case of CAA characterized at the molecular level.

##### Analbuminemia Bethesda

A single-base variant, C>T at position c.412 in exon 4 (c.412C>T) changes the codon CGA for p.Arg138 to stop codon TGA, resulting in premature termination and in a putative protein product of 113 residues (p.Arg138Ter). This molecular defect has been identified in two unrelated analbuminemic subjects: an American female (Watkins et al., 1994b), and a Swiss boy of non-consanguineous parents (Campagnoli et al., 2005a). The codon CGA is located in a CpG dinucleotide and a different variation (c.412C>G) produce albumin Yanomama (p.Arg138Gly), an alloalbumin present in polymorphic (>1%) frequency in an Amazonian Indian tribe (Watkins et al., 1994b).

##### Analbuminemia Seattle

A single G>A point variant at position c.714, the first base of exon 7 (c.714G>A), was found to cause CAA in a young Canadian male, who was diagnosed as analbuminemic in the neonatal period about 20 years earlier. This defect changes the codon TG/G for p.Trp238 to a stop codon TG/A, originating a putative protein product, p.Trp238Ter, of 213 amino acids (Watkins et al., 1994b).

##### Analbuminemia El Jadida

A G>T transversion at position c.802 in exon 7 (c.802G>T) converts the codon GAA for p.Glu268 to a stop codon TAA, resulting in a putative protein product of 243 residues, p.Glu268Ter. This defect was identified in two unrelated analbuminemic subjects: a 5-year-old Moroccan girl, the first child of a couple of consanguineous (first-degree cousins) parents (Campagnoli et al., 2005b), and in a 45-year-old man, born in Beirut, Lebanon, to consanguineous parents (first-degree cousins) of Syrian origin, living in Switzerland. The family comes from As Suwayda, a mainly Druze town located in Southwestern Syria, close to the border with Jordan (Dagnino et al., 2011).

##### Analbuminemia Roma-2

A C>T transition at position c.1225 in exon 10 (c.1225C>T) changes the codon CAG for p.Gln409 to a stop codon TAG, resulting in a putative protein product of 384 residues (p.Gln409Ter). This transition was found to cause CAA at the compound heterozygous state together with the Fondi defect (see below) in a 29-year-old Italian man born to apparently non-consanguineous parents (Campagna et al., 2005).

##### Analbuminemia Monastir

A C>A transversion at position c.1275 in exon 10 (c.1275C>A) was found to cause CAA in a 17-year-old Tunisian boy, son of apparently non-consanguineous parents. This variation changes the codon TAC for p.Tyr425 to a stop codon TAA, originating a predicted truncated polypeptide chain, p.Tyr425Ter, of 400 amino acids (Caridi et al., 2009).

##### Analbuminemia Tübingen

A C>T transition at position 1525 in exon 12 (c.1525C>T) was identified as the cause of the first two reported cases of CAA, two German siblings of a family with a high degree of consanguinity. This defect changes the codon CGA for p.Arg509 to a stop codon TGA, resulting in a putative protein, p.Arg509Ter, of 484 amino acids (Ruhoff et al., 2010).

#### Variants Affecting Splicing

##### Analbuminemia Baghdad

A G>A change of nucleotide c.79+1, the first base of intron 1 (c.79+1G>A), was shown to cause CAA in a newborn of Iraqi origin, first child of consanguineous (first-degree cousins) parents. This variant destroys the GT dinucleotide consensus sequence found at the 5′ end of most introns and causes the defective pre-mRNA splicing responsible for the analbuminemic trait. However, a search for the variant mRNA was not performed, and therefore the consequence of this defect at the protein level could not be evaluated (Campagnoli et al., 2002).

##### Analbuminemia Madeira

A single homozygous A>G transition at nucleotide c.138-2, the second last base of intron 2 (c.138-2A>G), was identified as the cause of CAA in a 40-year-old woman from Madeira Island (Portugal). The Madeira variant is expected to destroy the invariant AG dinucleotide sequence at the acceptor splice site of intron 2, causing a defective pre-mRNA splicing. However, a search for the variant mRNA could not be performed. The parents of the proband are first-degree cousins (Caridi et al., 2018a).

##### Analbuminemia Treves

An analbuminaemic infant of apparently non-consanguineous parents from Treves, Germany, was shown to be homozygous (and both parents heterozygous) for a G>T transversion at nucleotide c.270+1, the first base of intron 3 (c.270+1G>T). The variant inactivates the strongly conserved GT dinucleotide at the 5′ splice site consensus sequence of this intron, causing a defective pre-mRNA splicing. However, also in this case could a search for the variant mRNA not be performed (Caridi et al., 2016b).

##### Analbuminemia Zonguldak

A c.597T>A transversion in exon 5 (c.597T>A) was identified as the cause of CAA in a 3.5-month-old male Turkish patient of apparently non-consanguineous parents. This silent variant does not change the codon for p.Ala199, but creates at positions 597–598 near the 3′ end of exon 5 a new AG dinucleotide, the invariant sequence encountered in all eukaryotic intron acceptor splice sites. This aberrant splice site near the 3′ end of exon 5 might alter the normal splicing mechanism, although a search for the variant mRNA could not be performed (Caridi et al., 2008).

##### Analbuminemia Nijmegen-2

A single homozygous G>A transition at nucleotide c.615, the last base of exon 5, was identified as the cause of CAA in a man of Turkish descent, living in the Netherlands (c.615G>A). This silent variant does not change the codon for p.Lys205, but the alteration of the wild type donor site most probably affects splicing, even though a search for the variant mRNA could not be performed (Minchiotti and van der Burgt, unpublished results).

##### Analbuminemia Vancouver

A single A>G transition of the penultimate nucleotide at the 3′ end of intron 6, which inactivates the strongly conserved AG dinucleotide at the 3′ splice site consensus sequence (c.714-2A>G), was identified as the origin of CAA in a 12-year-old American Indian girl from remotely related parents. *In vitro*, the transition causes an undefined defect in splicing of the intron 6 sequence and the subsequent ligation of the exon 6-exon 7 sequences (Ruffner and Dugaiczyk, 1988).

##### Analbuminemia Guimarães

A G>A change at position c.1289+1, the first base of intron 10, inactivates the strongly conserved GT dinucleotide at the 5′ splice site consensus sequence of the intron (c.1289+1G>A) and causes the lack of ALB in a Portuguese boy from a couple of third degree cousins. This splicing defect results in the skipping of the preceding exon. The reading frame-shift in exon 11 produces a premature stop codon located 33 codons downstream from the 5′ end of the exon at amino acid position 430, giving rise to a putative protein product, p.Phe398Alafs^∗^33, of 405 amino acids (Caridi et al., 2012b). Later, the same splicing defect was found to cause the trait in 3 other analbuminemic individuals: an Algerian 24-year-old woman from a couple of first-degree cousins, and two young Turkish sisters, born from consanguineous (first-degree cousins) parents (Caridi et al., 2018b).

##### Analbuminemia Fondi

An A>G transition at nucleotide c.1427, the penultimate residue of exon 11 (c.1427A>G) introduces a novel, anticipated, GT donor splice site, in a consensus sequence GTGTGA. The subsequent reading frame-shift originates a premature stop codon 12 codons downstream in exon 12, giving rise to a putative protein product of 463 amino acids (p.Tyr476Serfs^∗^13). The Fondi defect was found to cause CAA at the compound heterozygous state (together with the allele Roma-2, see above) in a 29-year-old Italian man born to apparently non-consanguineous parents (Campagna et al., 2005).

##### Analbuminemia Tripoli

A G>T transition at nucleotide c.1428+1, the first base of intron 11 (c.1428+1G>T), was shown to cause CAA in two analbuminemic Libyan male infants from first-degree consanguineous parents. The transition was identified at the homozygous state in both children and in the heterozygous state in the parents and one of their daughters. It causes a defective pre-mRNA splicing, which is responsible for the analbuminemic condition, although its effect at the mRNA and at the protein level could not be studied (Bibi et al., 2012).

##### Analbuminemia Bartin

A single T>C transition at nucleotide c.1428+2, the second base of intron 11 (c.1428+2T>C), was shown to cause the lack of ALB in a 1-year-old Turkish analbuminemic female infant of apparently non-consanguineous parents. This defect, which inactivates the strongly conserved GT dinucleotide at the 5′ splice site consensus sequence of intron 11, results in the skipping of the following exon 11. The subsequent frame-shift within exon 12 originates a premature stop codon located 5 codons downstream, at position 411, giving rise to a predicted translation product, p.Leu431Tyrfs^∗^5, of 410 amino acids (Dolcini et al., 2007).

##### Analbuminemia Ankara

A homozygous G>A transition at position c.1652+1, the first base of intron 12 (c.1652+1G>A), which inactivates the strongly conserved GT dinucleotide at the 5′ splice site consensus sequence of this intron, was found to cause CAA in a Turkish newborn female of consanguineous (first-degree cousins) parents. The effect of this variant was evaluated by examining the cDNA obtained by reverse transcriptase-polymerase chain reaction from the ALB mRNA extracted from proband’s leukocytes. The splicing defect results in the complete skipping of the preceding exon (exon 12) and in a frame-shift within exon 13 with a premature stop codon after the translation of three variant amino acid residues. This extensive modification of the C-terminal region of ALB should give rise to a putative polypeptide chain of 455 amino acid residues, p.Leu477Cysfs^∗^4 (Caridi et al., 2014).

## Comments on Splicing Defects

The exon/intron junctions in the *ALB* conform with the invariant dinucleotides GT and AG consensus sequences present at the 5′ (donor) and 3′ (acceptor) splice sites, respectively (Mount, 1982; Minghetti et al., 1986). The most common consequence of splicing variants is skipping of one or more exons, followed by the activation of aberrant 5′ donor or 3′ acceptor splice sites and retention of full introns in mRNA (Vorechovsky, 2006). Of the 8 molecular defects located in consensus splicing regions and identified as cause of CAA, two (Madeira and Vancouver) are at acceptor splice sites, whereas six (Baghdad, Treves, Guimaraes, Tripoli, Bartin, and Ankara) are at donor splice sites, suggesting that the latter ones seem more prone to variants. In four cases (Bartin, Guimarães, Ankara, and Fondi), it was possible to isolate mRNA from white blood cells, and in all cases the splicing defects were verified at the mRNA level. Thus, these four splicing variants did not cause a complete degradation of the variant mRNA. In analbuminemia Bartin, Guimarães, and Ankara the inactivation of the GT dinucleotide at the 5′ splice site consensus sequence of an intron resulted in the complete skipping of the preceding exon and in a frame-shift within the following exon. For the other 3 variants destroying the GT dinucleotide consensus donor sequence, analbuminemia Baghdad, Treves, and Tripoli, the consequence of the splicing defect at the mRNA, and therefore at the protein level could not be evaluated. The same is true for the 2 molecular defects inactivating the invariant AG dinucleotide sequence at the acceptor splice site of an intron, namely analbuminemia Madeira and Vancouver. For the fourth defect studied at the mRNA level, analbuminemia Fondi, a variation near the 3′ end of an exon creates a novel, anticipated, GT donor splice site causing a subsequent reading frame-shift within the following exon. Finally, for analbuminemia Zonguldak the variant creates near the 3′ end of an exon a new AG dinucleotide sequence that was supposed to alter the normal splicing mechanism, whereas for analbuminemia Nijmegen-2 a variant in the last base of an exon alters the wild type donor site, most probably affecting splicing, but also in these cases the consequences of the splicing defects at the mRNA and protein level could not be studied.

In this context it is of interest to note that the presence of truncated ALB variants could never be evidenced in the serum of the analbuminemic individuals. One possible explanation for their absence could be due to the fact that all the putative aberrant albumin molecules are partially or totally lacking domain III, which has been shown to be crucial for ALB binding to the intracellular receptor FcRn. Normally, this binding mediates ALB rescue from lysosomal degradation and results in recycling of the protein back to the blood or to transcytosis (Sand et al., 2015). Alternatively, the lack of the variants may be caused by a nonsense-mediated decay of the altered transcript (Bhuvanagiri et al., 2010).

Variants affecting the GT consensus dinucleotide sequence at the donor intron splice sites in the *ALB* have also been reported to cause the presence of two genetic variants of the protein, albumins Rugby Park and Venezia (Kragh-Hansen et al., 2013; The Albumin Website, 2018). In albumin Rugby Park the alteration of the obligate GT sequence prevents splicing of intron 13 and translation continues for 21 nucleotides until a stop codon is reached. The extensive variant of albumin Venezia alters the first consensus nucleotide of the 5′ donor splice junction of intron 14 and the 3′ end of exon 14, which is shortened from 68 to 43 base pairs, causing an exon skipping event resulting in direct splicing of exons 13–15, which is normally untranslated. Interestingly, both these C-terminal variants were isolated from the sera of the heterozygous carrier individuals in levels ranging from 8 to 30% of the total albumin amount. Both variations are in the C-terminal end of the molecule: the new termination codons are located near the 3′ terminal exon of the mRNA, in intron 13 for albumin Rugby Park and in exon 15 for albumin Venezia. They could be recognized as proper by the nonsense-mediated mRNA decay pathway (Bhuvanagiri et al., 2010), or the proteins could have a sufficient binding to the intracellular receptor FcRn (Sand et al., 2015). In conclusion, of the eight variants in the *ALB* so far reported to alter the GT consensus sequence present at the 5′ exon/intron splicing sites, four (Bartin, Guimarães, Ankara, and albumin Venezia) resulted in skipping of the preceding exon, one (albumin Rugby Park) in retention of the intron in mRNA, whereas for Baghdad, Treves, and Tripoli it was not possible to establish the effect of the variant at the mRNA level.

## Conclusion

**FIGURE 4:**
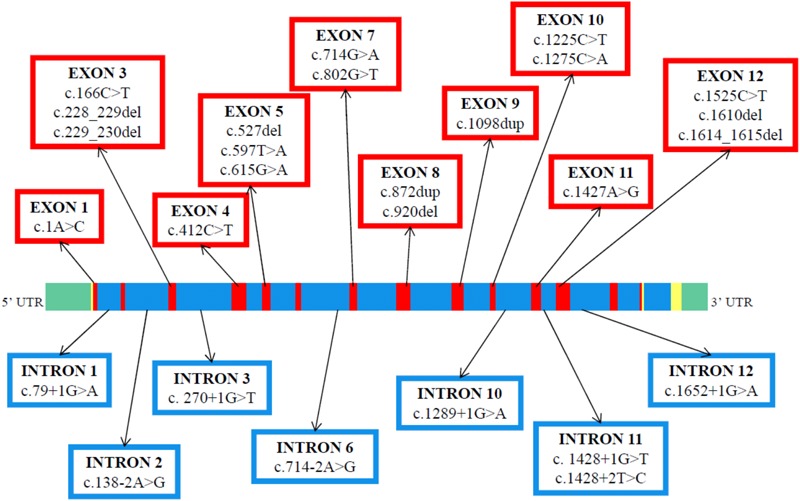
Genomic structure and distribution of variants resulting in CAA within the *ALB* gene. The linear map of the gene, the colors, and the symbols are as in Figure 1C. The map indicates the locations of 14 coding exons (red), the non-coding regions (yellow), the introns (blue) and the 5′ and 3′ untranslated region UTR (green). A summary of the reported variants is given in exon and intron-specific boxes. The variants are at the cDNA level (GenBank reference sequence: NM_000477.6).

Figure 4 shows that the 27 different molecular defects within the *ALB* so far reported to cause CAA are located in ten different exons (1, 3, 4, 5, 7, 8, 9, 10, 11, and 12) and in seven different introns (1, 2, 3, 6, 10, 11, and 12) (The Albumin Website, 2018; Caridi et al., 2019). Variations in the last two coding exons (13 and 14) would probably cause the presence of a circulating C-terminal variant of the protein and not CAA. The first twelve exons of *ALB*, with the exception of the two shortest, exons 2 and 6, were reported to contain at least one molecular defect resulting in CAA (The Albumin Website, 2018; Caridi et al., 2019). To outline a more accurate picture, nineteen variants are located within those ten exons, whereas the remaining eight variant sites were identified within seven introns (Figure 4). These findings seem to indicate that CAA is the result of widely scattered randomly occurring different molecular defects (Ruhoff et al., 2010).

**FIGURE 5:**
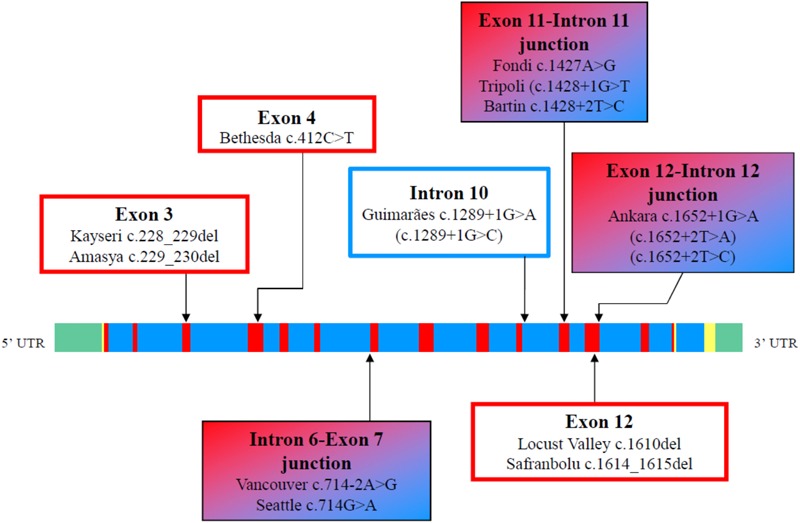
Regions in the *ALB* that seem to be more prone to variants resulting in CAA. The linear map of the gene, the colors, and the symbols are as in Figure 1C, 4. Variants at position c.412 in exon 4 may result in analbuminemia Bethesda (c.412C > T) or in bisalbumin Yanomama-2 (p.Arg138Gly; c.412C > G). These two variants are in a CpG sequence (c.412–413). LoF variants present in public database, but not yet identified as cause of CAA, are in parenthesis.

The majority of the causative defects found so far are unique, i.e., they were identified only in single individuals or within the same family. However, a few variants were identified in apparently unrelated, and geographically distant, individuals (The Albumin Website, 2018). These are the molecular defects known as Bethesda, El Jadida, Safranbolu, and especially Guimarães and Kayseri. The first 3 were each described in 2 apparently unrelated individuals (see above). The splicing defect c.1289+1G>A of analbuminemia Guimarães was reported in four analbuminemic individuals belonging to 3 apparently unrelated families (see above). Finally, the two base deletion c.228_229del (analbuminaemia Kayseri) is by far the most frequent cause of the trait, accounting for 14 of the 53 cases characterized at the molecular level (Table 1) and for about one third of the reported cases. By the way, the Kayseri defect represents also by far the most frequent variant found in heterozygosis in the average population among the 5 identified as cause of CAA and present in gnomAD and BRAVO (Table 3), representing about 2/3 of these cases. It is followed by the Guimarães defect, the ratio between the frequency of the Kayseri and that of the latter being about 4/1. Thus, the frequency of the more common variants so far identified as cause of CAA reflects their relatively high frequency in the average population. The very similar frequency of the Kayseri defect in the affected and in the control population could indicate the presence of a common ancestral allele. Moreover, the identification of the Kayseri defect in populations with very different geographical locations such as Native Americans (Saskatchewan), Turkish, Slovak gypsy and Swedish as well in control populations (gnomAD and BRAVO) suggests a possible founder effect; preliminary results utilizing SNPs analysis present in 2.5 kb of the *ALB* gene encompassing the Kayseri variant shows that all the analyzed samples share the same haplotype supporting the common ancestral allele hypothesis (Caridi, personal communication). However, the presence of the same defect in unrelated analbuminemic individuals might also indicate hypermutable regions in the gene. The regions in the *ALB* that seem to be more prone to variants resulting in CAA are summarized in Figure 5. These are:

• The region c.228–230 in exon 3, which includes the Kayseri and the adjacent Amasya variants.
• The position c.412 in exon 4. Variants at that position in a CpG sequence result in analbuminemia Bethesda and in albumin Yanomama-2.
• The intron 6-exon 7 junction (analbuminemia Vancouver and Seattle).
• The position c.1289+1 at the 5′end of intron 10 (analbuminemia Guimarães and 1 adjacent variant present in gnomAD which has not yet been identified as a cause of CAA).
• The exon 11-intron 11 junction (analbuminemia Fondi, Tripoli, and Bartin).
• The region c. 1610–1615 in exon 12 (analbuminemia Locust Valley and Safranbolu).
• The exon 12-intron 12 junction (analbuminemia Ankara and 2 adjacent variants present in gnomAD which have not yet been identified as causes of CAA).

In one case among the 53 characterized at the molecular level, case #32 of the Register of Analbuminaemia Cases (The Albumin Website, 2018), the variant could not be found within the *ALB* (Minchiotti, personal communication, confidential). Therefore, the possibility that the absence of ALB may be caused by variants in deep intronic regions of the gene, in remote regulatory elements, or by defects in the intracellular neonatal Fc receptor, FcRn, which binds and thereby prevents ALB from degradation in the lysosomes (Sand et al., 2015), cannot be ruled out.

An accurate follow-up of the known analbuminemic subjects, together with the identification of the molecular defects underlying new possible cases of CAA, will be necessary for a better understanding of the phenotype and the molecular genetics of this condition, which still have some dark sides.

## Author Contributions

LM, GC, MC, FL, MG, and UK-H made the literature research and wrote the manuscript. All the authors have read and approved the manuscript.

## Conflict of Interest Statement

The authors declare that the research was conducted in the absence of any commercial or financial relationships that could be construed as a potential conflict of interest.

## Footnotes

1 https://www.ncbi.nlm.nih.gov
